# The Contribution of Dietary Fructose to Non-alcoholic Fatty Liver Disease

**DOI:** 10.3389/fphar.2021.783393

**Published:** 2021-11-18

**Authors:** Siyu Yu, Chunlin Li, Guang Ji, Li Zhang

**Affiliations:** Institute of Digestive Diseases, Longhua Hospital, Shanghai University of Traditional Chinese Medicine, Shanghai, China

**Keywords:** non-alcoholic fatty liver disease (NAFLD), fructose, intestinal environment, *de novo* lipogenesis, inflammation

## Abstract

Fructose, especially industrial fructose (sucrose and high fructose corn syrup) is commonly used in all kinds of beverages and processed foods. Liver is the primary organ for fructose metabolism, recent studies suggest that excessive fructose intake is a driving force in non-alcoholic fatty liver disease (NAFLD). Dietary fructose metabolism begins at the intestine, along with its metabolites, may influence gut barrier and microbiota community, and contribute to increased nutrient absorption and lipogenic substrates overflow to the liver. Overwhelming fructose and the gut microbiota-derived fructose metabolites (e.g., acetate, butyric acid, butyrate and propionate) trigger the *de novo* lipogenesis in the liver, and result in lipid accumulation and hepatic steatosis. Fructose also reprograms the metabolic phenotype of liver cells (hepatocytes, macrophages, NK cells, etc.), and induces the occurrence of inflammation in the liver. Besides, there is endogenous fructose production that expands the fructose pool. Considering the close association of fructose metabolism and NAFLD, the drug development that focuses on blocking the absorption and metabolism of fructose might be promising strategies for NAFLD. Here we provide a systematic discussion of the underlying mechanisms of dietary fructose in contributing to the development and progression of NAFLD, and suggest the possible targets to prevent the pathogenetic process.

## Introduction

Nonalcoholic fatty liver disease (NAFLD) is characterized by an excessive fat build-up in the liver without clear other causes, e.g., alcohol addiction, virus infection, and drug induction. Epidemiology investigation reveals that NAFLD has affected more than one-quarter population around the world (Loomba and Sanyal, 2013; Softic et al., 2017). Recently, NAFLD is proposed to be named as metabolic (dysfunction) associated fatty liver disease (MAFLD) due to the heterogeneous etiology and metabolic risks (Eslam et al., 2020). NAFLD has a spectrum ranging from simple fatty liver (NAFL) to nonalcoholic steatohepatitis (NASH), related fibrosis, cirrhosis and even hepatocellular carcinoma (Williams et al., 2011). While NAFL is usually considered to be benign, patients with NASH often potentiate a high probability to further progression. Although NAFLD has been the focus of numerous studies, the pathological mechanisms are still unclear.

Fructose is a plant-derived monosaccharide, the natural form can be found in fruits, berries, and certain vegetables. The association of fructose with NAFLD can be derived from the ancient Egyptians, who fed ducks and geese-dried fruits to make foie gras. Industrial fructose, e.g., the high-fructose corn syrup, is widely used in almost all kinds of processed foods and beverages, partially driven by the huge commercial profits (Moeller et al., 2009; Vos and Lavine, 2013). During food processing, fructose undergoes a series of reactions (e.g., polymerization, condensation, *etc.*) upon heating to produce aldehydes, reducing ketones, and heterocyclic compounds, thus enhancing the flavor and improving the palatability of food (Maillard reaction). In the past 200 years, per capita dietary fructose intake has increased more than 100-fold (Jang et al., 2018). The average daily consumption of added sugars currently is estimated at 15% of total energy intake all over the world, and almost half is fructose (Wittekind and Walton, 2014; Newens and Walton, 2016; Powell et al., 2016). The wide application of processed foods, such as various baked food and yogurt, further exaggerate the increase of “invisible sugar” intake. Although fructose intake has significantly increased, the early warning mechanisms for the harm in humans are still immature. When excess glucose consumption raises blood glucose, insulin will be secreted to reduce the overwhelmed blood glucose, and prevents the continuous hyperglycemia. The metabolic rate of fructose is much higher than that of glucose, but no immediate feedback mechanisms to suppress its absorption or transportation. Furthermore, the transcription of glucose transporter 5 (GLUT5) increases upon fructose stimulation, which, in turn, enhances fructose transportation and absorption (Gouyon et al., 2003). Simultaneously, fructose is continuously transformed into fructose-1-phosphate (F1P) in the liver, which is an unrestricted process. High fructose-contained diets also induce more caloric intake, thus exacerbating the metabolic disorders indirectly (Lustig, 2013).

Fructose consumption is found to be positively correlated with obesity, diabetes, cardiovascular disease, NAFLD, hypertension, and cancer (Ouyang et al., 2008; Shapiro et al., 2011; White, 2013; DiNicolantonio et al., 2018). Excessive fructose-contained drinks intake is strongly related to the childhood obesity and pediatric NAFLD (Forshee and Storey, 2003). In adults ≥48 years old, daily fructose consumption increases hepatic inflammation and hepatocyte ballooning (Abdelmalek et al., 2010). By producing toxic advanced glycation end-products, fructose is also a threat to the aging process, the occurrence of diabetic complications (e.g. vascular, renal, and ocular complications), and the development of atherosclerosis (Gaby, 2005). In a prospective study involving 77,797 subjects in Sweden, overconsumption of high-sugar-containing foods is found to be a great risk of pancreatic cancer (Larsson et al., 2006). In addition, fructose is also the main cause of symptoms associated with chronic diarrhea or functional bowel disturbances (Gaby, 2005). In animals, overconsumption of fructose can assemble most metabolic features that associated with NAFLD patients, such as insulin resistance, hyperlipidemia, visceral obesity and hyperuricemia (Sánchez-Lozada et al., 2008), suggesting that fructose is a noticeable factor that drives the process of NAFLD. Therefore, we will review the recent studies that focus on the contribution of dietary fructose to NAFLD development and progression, and highlight the metabolic risks and possible drug targets.

## Dietary Fructose and Intestinal Environment

### Fructose and Dysbiosis

**FIGURE 1:**
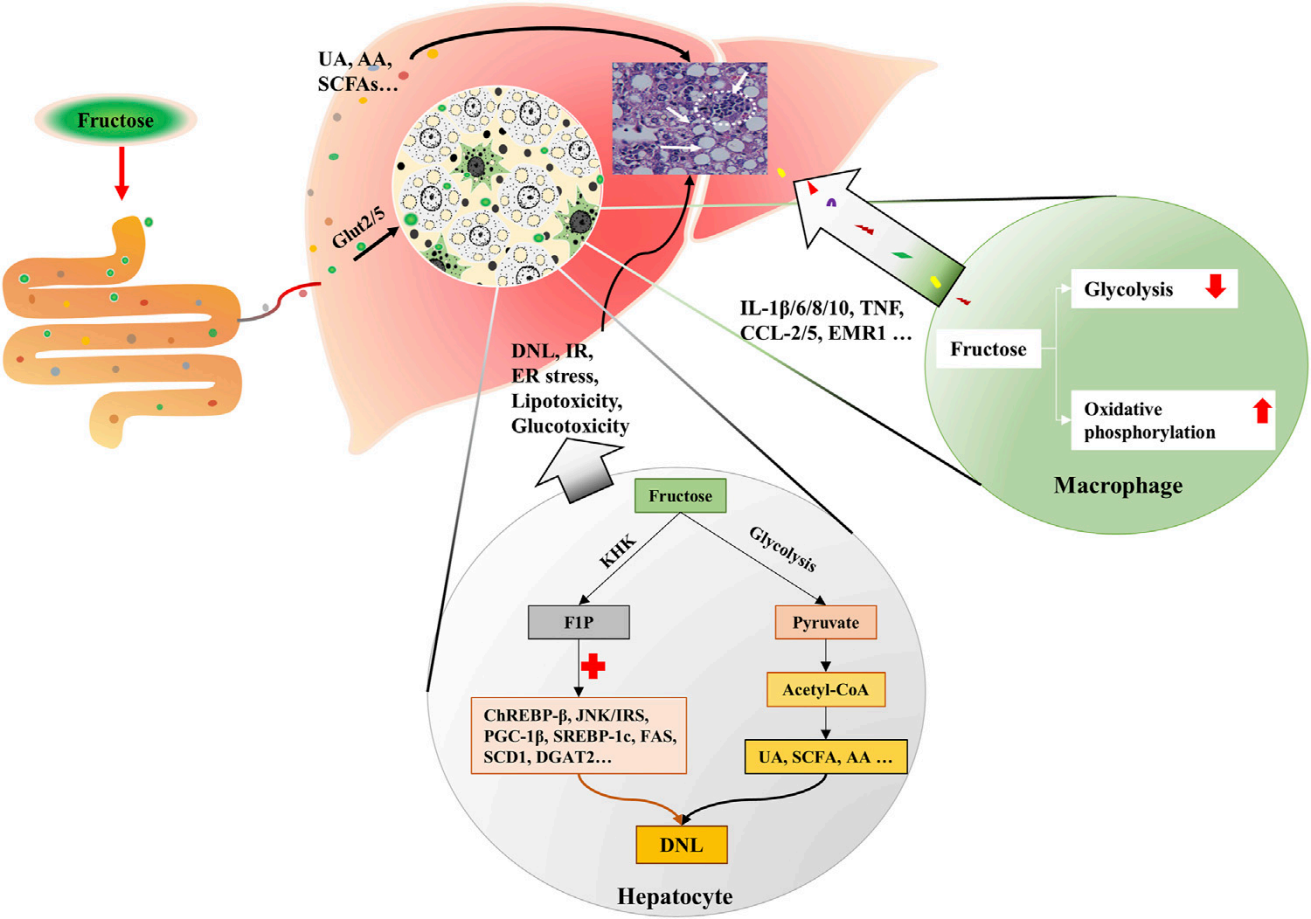
Excessive fructose intake leads to dysbiosis and gut barrier impairment. Fructose is absorbed in the small intestine, and transforms into glycerol, organic acids, and glucose, *etc.* by the action of resident microbiota. Fructose and fructose-derived metabolites may trigger dysbiosis characterized by decreased bacterial diversity and increased *Firmicutes*/*Bacteriodetes* ratio. Fructose over-intake also impairs gut barrier by decreasing protein expression of the tight junction (zonula occludens 1, occludin, claudin-1, and claudin-4) and adherent junction (β-catenin and E-cadherin) proteins, desmosome plakoglobin, and α-tubulin of enterocytes.

Diet is the main source of fructose, and most fructose is absorbed in the small intestine, with 25 g being the upper limit for a healthy adult. Fructose transport proteins (GLUT2 and GLUT5) that locate in the enterocytes are accounting for sensing and passively transporting fructose (Ferraris et al., 2018). Studies showed that GLUT5 transcription is specifically stimulated by fructose *via* cyclic adenosine monophosphate (cAMP) and phosphatidylinositol 3-kinase/protein kinase B (PI3K/Akt) systems (Cui et al., 2004; Cui et al., 2005), whereas GLUT2 is modulated by fructose and glucose levels, as well as systemic factors that released during their absorption (Cui et al., 2003). Absorbed fructose enters the liver *via* the portal vein for further metabolism. When fructose intake exceeds the absorptive capacity of enterocytes, it can transport to the colon where settles more than 100 trillion bacteria (Boulangé et al., 2016). These microbiomes are able to hydrolyze and ferment dietary polysaccharides by producing glycoside hydrolase enzymes (Xu et al., 2003; Sonnenburg et al., 2005; Patterson et al., 2016). By the action of gut microbiomes, fructose can transform into glucose, glycerol, and various organic acids, including uric acids (UA), short-chain fatty acids (SCFA) (e.g., acetate, butyric acid, butyrate and propionate), and amino acids (e.g., glutamic acid, glutamine and alanine) (Jahn et al., 2019; Qi et al., 2020; Zhao et al., 2020). These fructose metabolites either transport to the liver and serve as lipogenic substrates, or interact with the intestinal environment locally. As a monosaccharide, fructose can be the energy source for certain gut microbiomes, for instance, fructose is the single energy source of *Anaerostipes caccae DSM 14662* and *Roseburia intestinalis DSM 14610*, the acetate-converting and butyrate-producing strains (Falony et al., 2006). High fructose diet (HFrD) is reported to affect the composition of gut microbiota characterized by decreased bacterial diversity, increased *Firmicutes*/*Bacteriodetes* ratio in Sprague-Dawley rats (Sen et al., 2017), and reduced protective commensal and bile salt hydrolase-expressing microorganisms in dextran sodium sulfate (DSS)-induced colitis mice (Montrose et al., 2021). HFrD decreases the abundance of *Firmicutes* phylum (*Lactobacillus*) and *Verrucomicrobia* phylum (*Akkermansia*), while increases the abundance of *Bacteroidetes* phylum (*Bacteroides fragilis*) and *Proteobacteria* phylum (*Sutterella*, *Bilophila*, and *Escherichia*) in rodents (Sen et al., 2017; Zubiría et al., 2017; Do et al., 2018; Cho et al., 2021). And the alteration of these gut microbiomes might contribute to the dysbiosis and possibly the impairment of gut barrier (Figure 1). Luminal fructose at the physiological level stimulates the release of glucagon-like peptide 1 (GLP-1) from L-subtype EECs in humans and animals, however, the type 2 diabetic mice are GLP-1 resistant due to gut dysbiosis (Kuhre et al., 2014; Seino et al., 2015; Grasset et al., 2017).

### Fructose and Gut Barrier

When high-dose fructose saturates fructose clearance capacity in the ileum, the excessive fructose can enter the colon, and influence the gut barrier. Evidence implied that fructose acts on the intestinal barrier by regulating the transcriptional and post-translational modification of related proteins. In mammals, HFrD decreases protein expression of the tight junction (zonula occludens 1, occludin, claudin-1, and claudin-4) and adherent junction (β-catenin and E-cadherin) proteins, desmosome plakoglobin, and α-tubulin, and increases apoptotic proteins (p-JNK, Bax, cleaved caspase-3, and caspase-3 activity) of enterocytes (Cho et al., 2021). At the transcriptional level, prolonged HFrD feeding reduces genes encoding tight-junction protein-2, occluding, and different claudins in the intestine (Todoric et al., 2020) (Figure 1). In addition, HFrD feeding is reported to cause colon shortening possibly through endoplasmic reticulum (ER) stress and inflammatory reaction (Todoric et al., 2020). Recently, fructose is found to improve the survival of intestinal villus under hypoxia, resulting in the expanded nutrient absorption of the gut, thus contributing to increased body weight gain and fat accumulation in mice (Taylor et al., 2021). Nonetheless, it is still unclear whether there is an association between the extension of intestinal villi and disordered gut barrier.

Fructose also influences the intestinal barrier by regulating ethanol metabolism (Bradford et al., 1991; Uzuegbu and Onyesom, 2009; Villalobos-García et al., 2021). In alcohol-fed mice that lack alcohol dehydrogenase (ADH), fructose administration decreases the rates of ethanol metabolism by about 60% *via* diminishing H_2_O_2_ generation (Bradford et al., 1991). In light alcohol drinkers (<20 g/day) between 25 and 35 years old, however, fructose reduced the duration of alcohol intoxication by 30.7% through facilitating its clearance (Uzuegbu and Onyesom, 2009). At the same time, dysbiosis in fructose-exposed rodents may contribute to the endogenous ethanol production. Additionally, excess fructose intake may lead to fructose malabsorption, and unabsorbed fructose has been found to draw fluid into the intestinal lumen, resulting in abdominal pain, flatulence, diarrhea, and other digestive dysfunctions (Beyer et al., 2005; Gaby, 2005). The organic acids and gas that are produced during fructose metabolism also contribute to the aforementioned gastrointestinal symptoms (Southgate, 1995; Beyer et al., 2005).

## Fructose Metabolism in the Development of NASH

### Fructose Metabolism and Hepatic Lipogenesis

Fructose dose as low as 0.33 g·kgBM^−1^ is sufficient to stimulate *de novo* lipogenesis (DNL) in mice (Tran et al., 2010), while fructose <0.35 g·kgBM^−1^ would not appear in the portal blood as a prototype (Jang et al., 2018), implicating that gut-derived fructose metabolites, e.g., acetate, amino acid, endotoxins, SCFA, and ethanol can enter the liver and stimulate lipogenesis physiologically. The saturation of intestinal fructose catabolism would occur at about 5 g sugar intake (e.g., one-fourth of a banana) (Ferraris et al., 2018; Gonzalez and Betts, 2018). And the hepatocellular metabolism of fructose (which bypasses the rate-limiting step of glycolysis at the level of phosphofructokinase) is responsible for most of the metabolic disorders (Andres-Hernando et al., 2020). Fructose at the physiological level can be completely metabolized by the liver, overwhelming fructose consumption, however, leads to steatosis *via* promoting lipogenesis, dyslipidemia, visceral adiposity, and insulin resistance (Jin and Vos, 2015). In addition, fructose metabolism lacks hormonal regulation (Douard and Ferraris, 2008; Douard et al., 2013), which may explain the significant variation in peripheral fructose levels (from 0.008 to 16 mM) (Kawasaki et al., 2002; Hui et al., 2009). On the contrary, fructose malabsorption is negatively correlated to hepatic steatosis (Walker et al., 2012), suggesting that fructose is an environmental factor that favors the development of NAFLD.

**FIGURE 2:**
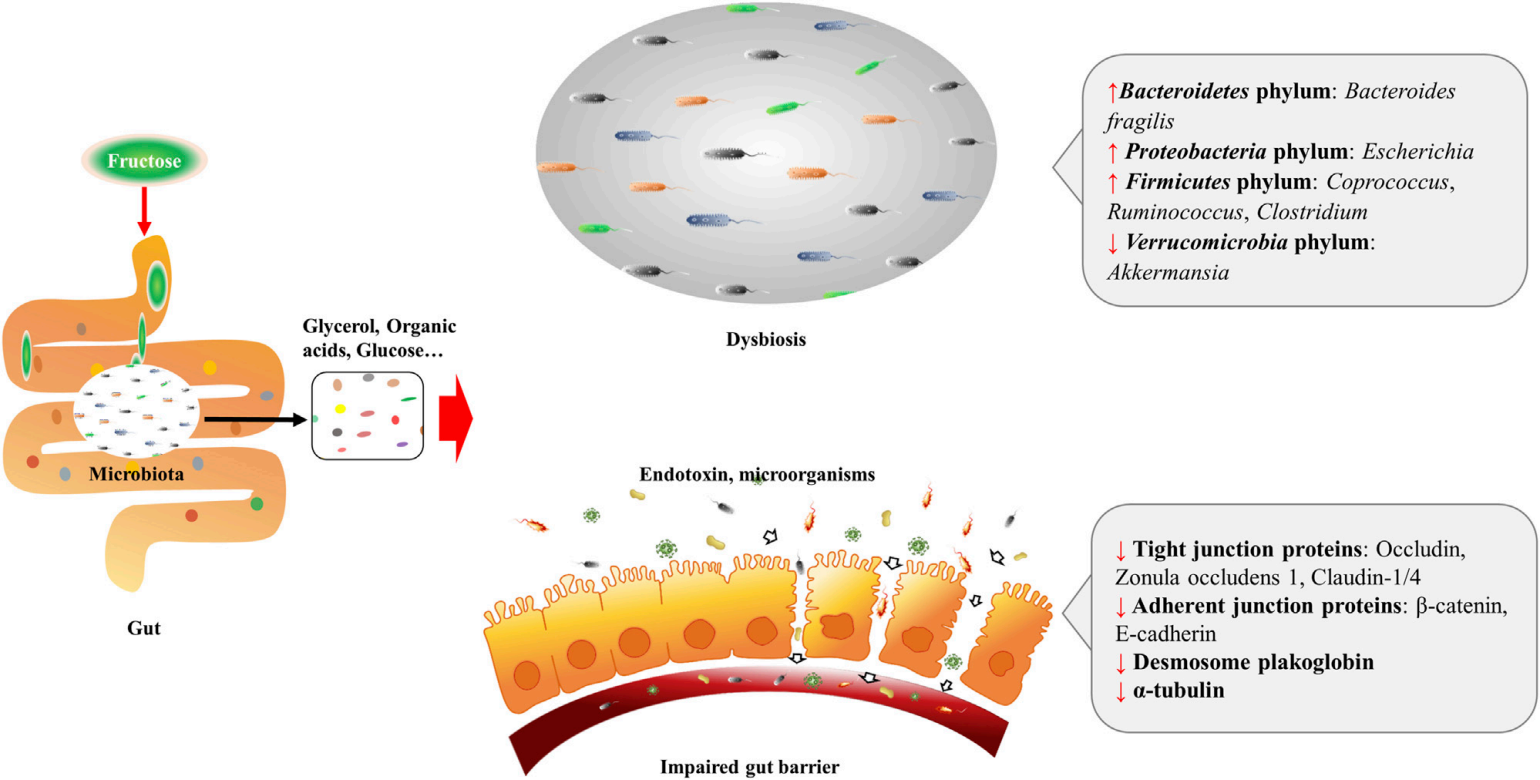
Fructose metabolism in the liver. Fructose is mainly catabolized by hepatocytes. Fructose is absorbed by hepatocytes *via* Glut2/5. Intracellular fructose transforms into F1P by the action of KHK. Then, F1P is metabolized into pyruvate and acetyl-CoA for tricarboxylic acid cycle. During this process, fructose, F1P, as well as their intermediates can activate ChREBP-β, JNK/IRS, and PGC-1β/ SREBP-1/ACC1, which results in DNL. Gut-derived fructose metabolites enter the liver through portal vein, and contribute to DNL in the liver. In addition, UA, SCFA and AA produced in this process also trigger DNL. Simultaneously, fructose exposure alters the energy supply mode of macrophages, which induces oxidative stress and secretion of inflammatory cytokines. KHK, fructokinase; F1P, fructose-1-phosphate; ChREBP-β, carbohydrate-responsive element-binding protein β; JNK, c-Jun N-terminal kinase; IRS, insulin receptor substrates; PGC-1β, peroxisome proliferator-activated receptor gamma co-activator-1β; SREBP-1, sterol regulatory element-binding protein-1; ACC1, acetyl-CoA carboxylase 1; DNL, *de novo* lipogenesis; UA, uric acid; SCFA, short chain fatty acid; AA, amino acid.

More than a quarter of the intrahepatic lipid is generated by DNL in obese and NAFLD individuals (Donnelly et al., 2005; Lim et al., 2010). Fructose enters hepatocytes in a GLUT5-mediated process, intracellular fructose transforms into Fructose-1-phosphate (F1P) by the action of fructokinase (KHK) (Mirtschink et al., 2018). Then, F1P is metabolized into pyruvate and acetyl-CoA for the tricarboxylic acid cycle (Lustig, 2013). During this process, fructose, F1P, as well as their intermediates can activate carbohydrate-responsive element-binding protein (ChREBP)-β, c-Jun N-terminal kinase/insulin receptor substrates, and peroxisome proliferator-activated receptor gamma co-activator-1β/sterol regulatory element-binding protein-1(SREBP-1)/acetyl-CoA carboxylase 1 (ACC1) and downstream DNL pathways (Lustig, 2013; Premachandran et al., 2017; Softic et al., 2017; Gonzalez and Betts, 2018; Pan et al., 2018). Concurrently, genes that encode fatty acid oxidizing enzymes are down-regulated upon fructose exposure, which further exacerbate the hepatic lipid accumulation (Premachandran et al., 2017; Pan et al., 2018). The metabolites of fructose in the intestine, such as SCFA, and amino acid also provide substrates for hepatic DNL, leading to the lipid accumulation in hepatocytes and the increase of circulating fatty acids (Jang et al., 2018; Jahn et al., 2019; Zhao et al., 2020) (Figure 2). Besides, fructolysis provides a carbon backbone for the synthesis of nucleotides, triglyceride-glycerol (glycerol-3-phosphate), and amino acids (serine, glutamate, glutamine, aspartate, and asparagine) (Liu et al., 2020).

Fructose in the liver can be transformed into glycogen (Davies et al., 1990; Warner et al., 2021), and hepatic glycogen storage also stimulates DNL (Hengist et al., 2019). In humans, fructose intake rapidly increases post-exercise liver glycogen synthesis (Décombaz et al., 2011). In rats, administration of fructose causes an immediate increase of glycogen synthase (GS) activity and initiates a fast accumulation of glycogen in the liver (Bezborodkina et al., 2014). Actually, glucose is usually used in food processing in a ratio of nearly 1:1 with fructose, high levels of glucose in the portal vein can induce the expression of hepatic aldose reductase (AR), resulting in the conversion of glucose into sorbitol, which is then metabolized to fructose by sorbitol dehydrogenase (Lanaspa et al., 2013). The production of endogenous fructose may further increase the total fructose content. Fructose contributes to hepatic lipid accumulation also by blocking fatty acid oxidation (FAO) (Lanaspa et al., 2012a; Lanaspa et al., 2012b). In high-fat high-fructose-fed mice, the activity of CTP1a (the rate-limiting enzyme of FAO) and its acylcarnitine products are decreased, and the mitochondrial fission and mitochondrial integrity are damaged (Softic et al., 2019), suggesting the blockage of FAO.

### Fructose and Liver Inflammation

Fructose is believed to be an inflammatory mediator that promotes the progression of NAFLD to NASH (Jegatheesan and De Bandt, 2017). HFrD has been proven to induce insulin resistance, hepatic steatosis, ballooning degeneration and fibrosis in transgenic (Tg) MUP-uPA mice, and significantly increased the tumor necrosis factor (*Tnf*), interleukin (*Il*)-*6*, *Il1β*, *Ccl2*, *Ccl5* and *Emr1* (Todoric et al., 2020). Moreover, HFrD enhances diethyl nitrosamine-induced hepatocellular carcinoma in BL6 mice (Todoric et al., 2020).

Macrophage plays an important role in the hepatic inflammatory response. Fructose is thought to influence the metabolic phenotype of macrophages, which is a decisive factor for cellular function. Stressed macrophages present increased glycolysis, whereas oxidative metabolism primed macrophages for a less inflammatory mode (Vats et al., 2006). 2-deoxy-glucose (2-DG), an inhibitor of glycolysis, also blocks TNF-α and IL-6 production in macrophages (Wang et al., 2014). In lipopolysaccharide (LPS)-stressed macrophages, high concentrations of fructose alter the mode of energy supply, with impaired glycolysis and enhanced oxidative phosphorylation, along with glutaminolysis and oxidative stress, which result in the secretion of inflammatory cytokines, such as interleukin-1β (IL-1β), IL-6, IL-8, IL-10, and TNF (Jones et al., 2021) (Figure 2). Considering the fact that increased glycolysis favors cell proliferation in cancer while enhanced oxidative phosphorylation supplies sufficient amounts of energy for inflammatory macrophages, the different effects of fructose on cells may be derived from cell demands in different situations. And glycolysis-produced intermediates may contribute to both cell proliferation and activation.

And fructose overconsumption is shown to induce local macrophage infiltration in the liver (Hotamisligil et al., 1993; Glushakova et al., 2008; DiNicolantonio et al., 2018). Studies also show that fructose promotes monocyte recruitment *via* monocyte chemoattractant protein 1 and intracellular adhesion molecule 1 (Fantuzzi and Faggioni, 2000; Shapiro et al., 2011; DiNicolantonio et al., 2018). Besides, fructose exposure in LPS-stimulated human dendritic cells provokes the secretion of pro-inflammatory cytokines from T cells (Jaiswal et al., 2019). And mice deficient in T or NK cells are prevented from developing fructose-induced NAFLD (Bhattacharjee et al., 2014). Moreover, fructose treatment influences the viability of stressed immune cells (Jones et al., 2021).

Fructose overconsumption also increases visceral adipose tissue mass and intracellular cortisol concentration (Targher et al., 2006; Kovačević et al., 2014). Adipocyte hypertrophy may trigger ER stress, disturb adipokines (leptin and adiponectin) release, and increase pro-inflammatory cytokine secretion (de Heredia et al., 2012; DiNicolantonio et al., 2018). Fructose-induced increase of intracellular cortisol level *via* 11beta-hydroxysteroid dehydrogenase type1 (11β-HSD1) leads to a raise of fatty acid flux out of the subcutaneous adipocytes, thus providing more substrates for visceral fat accumulation (de Heredia et al., 2012; DiNicolantonio et al., 2018). Plasminogen activator inhibitor 1 (PAI-1) is an acute-phase protein that participates in hepatic lipid transport and provokes inflammation in the liver (Bergheim et al., 2006). It is reported that fructose content is positively correlated to PAI-1 concentration, and PAI-1 knock-out mice are protected from fructose-induced steatosis and inflammation (Castrogiovanni et al., 2012).

## Dietary Fructose and UA Metabolism

Dietary fructose increases both serum and intrahepatic UA levels (Lanaspa et al., 2012b). UA is primarily produced in hepatocytes, and the process of fructose metabolization to F1P consumes a large amount of intracellular ATP and phosphate, the decrease of phosphate activates adenosine monophosphate (AMP) deaminase that converts AMP to inosine monophosphate, which results in UA production (Van den Berghe, 1986). Simultaneously, fructose-derived amino acids also increase UA excretion and decrease plasma uridine (Yamamoto et al., 1999). UA is known to promote hepatic fat accumulation, and provokes mitochondrial oxidative stress by increasing superoxide generation and mitochondrial NOX4 expression, and decreasing manganese superoxide dismutase in NASH mice (Lanaspa et al., 2012b; Ishimoto et al., 2012). Meanwhile, UA can activate nuclear factor-κB and Nod-like receptor protein 3 inflammasomes (Wan et al., 2016). In patients with NAFLD/NASH, the serum UA level is positively correlated to the extent of the lobular inflammation and steatosis (Sertoglu et al., 2014). Additionally, UA may increase endogenous fructose production in a feedback way (Jensen et al., 2018). Inhibition of UA formation has been proven to be beneficial for metabolic diseases (Lanaspa et al., 2012c; Lanaspa et al., 2013; Nakatsu et al., 2015).

Fructose metabolites are found to induce renal damage (Ramezani and Raj, 2014; Tang et al., 2015). In healthy male adults, daily ingestion of 200 g fructose for 2 weeks appears to increase urinary stone formation partly *via* regulating urate metabolism, urinary pH, and increasing oxalate (Johnson et al., 2018). Fructose-contained beverage intake in infancy has been reported to be associated with worse outcomes in a later event of acute kidney injury and kidney damage during adolescence (García-Arroyo et al., 2020). In experimental hamsters, monosodium glutamate plus a high-fat and high-fructose (HFF) diet increases the risk of kidney injury, induces gut dysbiosis, and an increase in the amount of p-cresol sulfate (Pongking et al., 2020). At the same time, HFF can induce dyslipidemia and lipid accumulation in the kidney (Thongnak et al., 2020). Besides, fructose increases segmental artery vascular resistance by increasing serum UA and copeptin (Chapman et al., 2020), enhances angiotensin II-stimulated Na transport *via* activation of protein kinase C α1 in renal proximal tubules (Yang et al., 2020). Renal DNL stimulated by high-fructose supplementation may also contribute to the increase of intrahepatic triglycerides (Milutinović et al., 2020).

## Possible Drug Targets in Fructose Metabolism

### Glucose Transporter Family Members (GLUTs)

Glucose transporter family members (GLUTs) belong to the major facilitator superfamily, which are encoded by the SLC2 genes (Mueckler and Thorens, 2013; Nomura et al., 2015). In humans, 14 GLUTs are found to mediate the facilitative diffusion of sugar along the concentration gradient (Schmidl et al., 2020). Dietary fructose absorption is very efficient in the small intestine mediated by GLUTs transporters.

The transport of fructose across the intestinal basolateral membrane is mediated by one or more sodium-independent routes. GLUT2 is responsible for moving both fructose in and glucose out of the enterocyte across the basolateral membrane under basal conditions (Cheeseman, 1993). The malfunction or dysregulation of GLUT2 is associated with diabetes, metabolic syndrome, and cancer (Schmidl et al., 2020). GLUT2 variants increase the risks of fasting hyperglycemia, type 2 diabetes, hypercholesterolemia and cardiovascular diseases. Besides, individuals with a missense mutation in GLUT2 show a preference for sugar-containing foods (Thorens, 2015). GLUT2 inhibition is desirable for patients with fructose-induced metabolic disorders, but it may lead to insulin insensitivity (Ferraris et al., 2018), suggesting that specific inhibition of GLUT2 is necessary. GLUT5 level is increased in patients with acute myeloid leukemia and prostate cancer, and is reported to be negatively related to the prognosis of patients (Carreno et al., 2021; Jeong et al., 2021). In rodents, increased GLUT5 is associated with enhanced steatosis and pancreatic inflammation (Roncal-Jimenez et al., 2011). GLUT5 deletion could reduce fructose absorption by approximately 75% in the jejunum and decrease the concentration of serum fructose by approximately 90% in comparison to wild-type mice on excess fructose intake, suggesting that GLUT5 is required for fructose transportation, and the function cannot be compensated by GLUT2 (Barone et al., 2009; Patel et al., 2015). GLUT5 is also highly expressed in many cancers, thus promoting the proliferation of cancer cells (Su et al., 2018; Jin et al., 2019; Chen et al., 2020). Inhibiting GLUT5 is expected to be a promising strategy for fructose-associated diseases. Lately, several drugs are found to inhibit GLUT2/5. *i.e.*, Chamomile and green tea display acute inhibition on GLUT2/5 and thus decrease fructose and glucose transportation in human Caco-2 cells and Xenopus oocytes (Villa-Rodriguez et al., 2017). Kefir, a fermented drink, is found to decrease GLUT2/5 in the liver of HFrD fed rats (Akar et al., 2021).

### Fructokinase (KHK)

KHK is an essential fructose-metabolizing enzyme in the liver, which specifically catalyzes the transfer of a phosphate group from adenosine triphosphate to fructose. KHK-C, the principal isoform of KHK in the liver, is increased in NAFLD patients that consume excessive fructose-contained beverages (Ouyang et al., 2008; Ishimoto et al., 2012). Pharmacological inhibition of KHK activity with PF-06835919 is reported to prevent patients from hepatic steatosis, lipogenic, and fibrosis (Shepherd et al., 2021). KHK deficiency or inhibition (PF-06835919) protects animals from fructose-induced obesity, insulin resistance, hypertriglyceridemia, and NAFLD (Lanaspa et al., 2013; Futatsugi et al., 2020; Gutierrez et al., 2021). Knockout KHK could also decrease fructose-derived UA production and attenuate mitochondrial oxidative stress in mice (Ishimoto et al., 2012). Besides, Extracts of Angelica archangelica, Garcinia mangostana, Petroselinum crispum, and Scutellaria baicalensis are identified with inhibitory activity against ketohexokinase-C (Le et al., 2016). Interestingly, fructokinase A (KHK-A), another isoform of KHK, protects against C-mediated metabolic diseases, but its activity is relatively low due to a low fructose affinity (Ishimoto et al., 2012). Drugs such as that targeting reshaping the balance between the two types of KHK is an attractive strategy.

### Triokinase (TK)

TK is a rate-limiting enzyme in fructolysis, and is essential for the induction of the lipogenic program under physiological conditions (Liu et al., 2020). TK knockdown abrogates the expression of lipogenic enzymes and results in a significant reduction in HFrD-induced hepatic steatosis, as well as liver size and plasma glucose levels (Liu et al., 2020). Meanwhile, TK is necessary for maintaining mitochondrial respiration. It is found that 5 mM fructose is sufficient to have a significant decapacitating effect on mitochondrial respiration in the TK-deficient cells, an overnight culture of mutant cells with 15 mM fructose obliterates their respiration capacity (Liu et al., 2020). Liver TK deficiency results in a more than 2-fold sensitization of hepatocytes to fructose toxicity, indicating TK might be a promising target to block fructose toxicity. Although ADP is reported to be a potent inhibitor of TK, agents that target TK inhibition are not available yet.

### Aldose Reductase (AR)

AR is the key enzyme in stimulating endogenous fructose production. It can be activated under pathological conditions, including ischemia, heart failure, inflammation, and hyperuricemia (Huang et al., 2017; Nakagawa et al., 2020). Increased AR results in the conversion of blood glucose into sorbitol, which is further metabolized into endogenous fructose by sorbitol dehydrogenase (Lanaspa et al., 2013). Liver AR knockdown, or AR inhibitors can attenuate hepatic steatosis and related metabolic disorders in mice (Lanaspa et al., 2013; Nakagawa et al., 2020). Furthermore, the addition of salt to fructose in the drinking water significantly accelerates the development of NAFLD, the high-salt diet activates the AR-fructokinase pathway in the liver and hypothalamus, resulting in endogenous fructose production (Lanaspa et al., 2018). These results suggest that AR is the target of blocking endogenous fructose production. Actually, a series of extracts from natural products such as Luteolin, quercetin, apigenin, fisetin, and myricitrin are potential AR inhibitors, and found to be beneficial for NAFLD/NASH (Grewal et al., 2016). And AR inhibitors Sorbinil and Ranirestat, are under clinical trials.

## Conclusions and Perspectives

The liver is the critical organ to metabolize fructose, the increasing consumption of fructose in various forms specially exaggerates liver burden and contributes to NAFLD/NASH. The rapidly metabolized fructose in the liver promotes lipogenesis, lipotoxicity, as well as the inflammatory reaction of immune cells. Meanwhile, the interaction of fructose and gut microbiota results in dysbiosis, impaired intestinal mucosa barrier, production of toxins and microbial metabolites that may further serve as substrates for liver lipogenesis, and pathogens for liver inflammation. In addition, fructose metabolism lacks hormonal regulation, making excessive fructose consumption thus a more dangerous factor to NAFLD patients.

Even so, patients with obesity, hypertension, and diabetes are encouraged to intake certain fructose contained in vegetables and specific fruits, such as blueberries, grapes, and apples (Bazzano et al., 2008; Muraki et al., 2013; Sundborn et al., 2019). The possible explanation might be that most fruits contain modest amounts of fructose (3–8 g per fruit), and the fiber, vitamin, and other constituents (flavonols, epicatechin, ascorbate, and other antioxidants) in it carries substantial metabolic benefits (Vasdev et al., 2002; Sundborn et al., 2019). Restriction of calorie intake when supplying high fructose failed to induce obesity but still triggered steatosis in rats (Roncal-Jimenez et al., 2011). Even supplement with high palm oil in high fructose diet, the rats tend to become non-obese NAFLD model (Abdelmoneim et al., 2021), suggested fructose induction may account for lean NAFLD. Lean NAFLD makes up about one-third of the NAFLD population, according to recent studies, lean NAFLD may potentiate higher metabolic risks than obese NAFLD. Thus, the association of fructose intake and lean NAFLD deserve to be systematically explored. Also, the fructose-associated NAFLD in children and adolescents needs to be highlighted. Considering the global epidemic of metabolic syndrome especially NAFLD, it is extremely important to provide alarming in controlling daily fructose intake for people, especially children and teenagers, in prevention and management of NAFLD.
